# Retraction: Plant-growth-promoting *Bacillus* and *Paenibacillus* species improve the nutritional status of *Triticum aestivum* L.

**DOI:** 10.1371/journal.pone.0294880

**Published:** 2023-11-16

**Authors:** The PLOS ONE Editors

The *PLOS ONE* Editors retract this article [1] because it was identified as one of a series of submissions for which we have concerns about competing interests and peer review. In addition, the editorial team has serious concerns about a number of citations included in this article, including concerns of extensive self-citation and inclusion of inappropriate citations for statements in the article. We regret that the issues were not addressed prior to the article’s publication.

The *PLOS ONE* Editors note that primary data were not provided with the article, contrary to the Data Availability statement.

AH, MA, MN, ZI, ML, AM, and JTC did not agree with the retraction. MJ, FMP, SA, MNA, and PA either did not respond directly or could not be reached.
